# Profile of gene expression changes during estrodiol-17β-induced feminization in the *Takifugu rubripes* brain

**DOI:** 10.1186/s12864-021-08158-0

**Published:** 2021-11-24

**Authors:** Xufang Shen, Hongwei Yan, Jieming Jiang, Weiyuan Li, Yuyu Xiong, Qi Liu, Ying Liu

**Affiliations:** 1 College of Life Sciences, Liaoning Normal University, Dalian, 116029 Liaoning China; 2 Key Laboratory of Environment Controlled Aquaculture (Dalian Ocean University) Ministry of Education, Dalian, 116023 China; 3 College of Fisheries and Life Science, Dalian Ocean University, Dalian, 116023 Liaoning China; 4 College of Marine Science and Environment Engineering, Dalian Ocean University, Dalian, 116023 Liaoning China

**Keywords:** *Takifugu rubripes*, Brain, Estradiol-17β, Sex differentiation, Transcriptome analysis

## Abstract

**Background:**

As the critical tissue of the central nervous system, the brain has been found to be involved in gonad development. Previous studies have suggested that gonadal fate may be affected by the brain. Identifying brain-specific molecular changes that occur during estrodiol-17β (E_2_) -induced feminization is crucial to our understanding of the molecular control of sex differentiation by the brains of fish.

**Results:**

In this study, the differential transcriptomic responses of the *Takifugu rubripes* larvae brain were compared after E_2_ treatment for 55 days. Our results showed that 514 genes were differentially expressed between E_2_-treated-XX (E-XX) and Control-XX (C-XX) *T. rubripes*, while 362 genes were differentially expressed between E_2_-treated-XY (E-XY) and Control-XY (C-XY). For example, the expression of *cyp19a1b*, *gnrh1* and *pgr* was significantly up-regulated, while *st, sl, tshβ, prl* and *pit-1,* which belong to the growth hormone/prolactin family, were significantly down-regulated after E_2_ treatment, in both sexes. The *arntl1*, *bhlbe*, *nr1d2*, *per1b*, *per3*, *cry1*, *cipc* and *ciart* genes, which are involved in the circadian rhythm, were also found to be altered. Differentially expressed genes (DEGs), which were identified between E-XX and C-XX, were significantly enriched in neuroactive ligand-receptor interaction, arachidonic acid metabolism, cytokine-cytokine receptor interaction and the calcium signaling pathway. The DEGs that were identified between E-XY and C-XY were significantly enriched in tyrosine metabolism, phenylalanine metabolism, arachidonic acid metabolism and linoleic acid metabolism.

**Conclusion:**

A number of genes and pathways were identified in the brain of E_2_-treated *T. rubripes* larvae by RNA-seq. It provided the opportunity for further study on the possible involvement of networks in the brain-pituitary-gonadal axis in sex differentiation in *T. rubripes*.

**Supplementary Information:**

The online version contains supplementary material available at 10.1186/s12864-021-08158-0.

## Background

Sex determination and differentiation are the most essential processes for species reproduction [1]. Sex determination is defined as the developmental process by which the sex is established. Gonadal sex differentiation is defined as the process during which the undifferentiated gonad develops into either an ovary or a testis after the determination of sex [2]. Since sexual dimorphism (such as body size and growth rate) is common in fish, elucidating the mechanism involved in sex determination and differentiation, which may lead to the development of a sex control technique, is of great commercial interest in the aquaculture industry [3]. Moreover, as the largest group of vertebrates, fish display the greatest diversity of sexual phenotypes and are considered excellent models for the investigation of mechanisms involved in sex determination, differentiation, and sexual plasticity. Unlike mammals, sex determination and differentiation are tremendously complex and flexible in fish and the sexual fate of fish has been proven to be affected by exogenous factors (such as social dynamics, temperature, light conditions, density, pH, stress and hormones) [3–6]. Among those factors, estrogens are conserved and are known to be required for ovarian differentiation and maintenance of the female phenotype [6]. Prior to sexual differentiation, the administration of estrodiol-17β (E_2_) can induce sex reversal (male-to-female) in fish [7, 8]. In fish, estrogens are synthesized by the aromatization of androgens, through cytochrome P450 aromatase, which is mainly encoded by *cyp19a1a/b* [9]. Previous studies have shown that treatment with an aromatase inhibitor and knockout of *cyp19a1a* can result in sex reversal in the female [10–12]. E_2_ treatment methods have been widely applied to sex ratio control, in particularly with respect to establishing a monosex population to understand the roles of endocrine and genetic factors regulating sex determination and differentiation in academic research [13–16].

*Takifugu rubripes*, which is commonly known as the tiger puffer or torafugu, is one of the most popular marine-cultured species in Asia. It is famous for its umami taste and has been available in local Chinese markets since 2016. Tiger puffer aquaculture is mainly distributed across the north coast of China, and the production of farmed pufferfish was over 10, 000 metric tons in 2020 (data from China Fisheries Statistics 2021). More than 90% of farmed tiger puffers are exported to Japan and South Korea. Since *T. rubripes* testis is a delicacy and ovaries quite poisonous, male torafugu are more expensive and popular than female torafugu. Hence, monosex male production is desirable in aquaculture. Moreover, *T. rubripes* is considered as an ideal model for investigating the molecular mechanisms that underlie sex determination and differentiation as it has a relatively small and compact genome, when compared to other vertebrates [17, 18].

*T. rubripes* is a gonochoristic fish and with an XX/XY sex determination system and an allelic variation in the *amhr2* gene (the AMH receptor) that has been shown to be responsible for maleness [18]. The process of gonadal development in *T. rubripes* and the expression profiles of genes related to the sex differentiation process have been described in previous studies [19–21]. Intercrosses between E_2_-induced generate pseudo female and normal males resulted in the supermale (YY). In *T. rubripes*, YY males can be used to produced monosex male for the study of gonadal sex differentiation and increasing interest in commercial production. Although whether the sex reversed XY or XX torafugu are fertile or not has not been confirmed until now, it has been demonstrated that treatment of XX *T. rubripes* with aromatase inhibitor (fadrozole or letrozole) results in the inhibition of ovarian cavity formation [8, 20, 22]. Treatment of genetically XY *T. rubripes* with E_2_, prior to morphological sex differentiation, can induce feminization. Several genes involved in E_2_-induced feminization in the gonads of *T. rubripes* have been characterized in our previous study [22].

Prior to differentiation of the gonads, sexual differences exist in non-gonadal tissues as well as the germline. Thus, these differences are created up-stream of gonadal differentiation [23–25]. As the critical tissue of the central nervous system, the brain has been found to be involved in germline development, and the differential development of the two sexes could be the result of differential gene expression in the brain, prior to the formation of the gonads [24–29]. Several sexually dimorphic markers or genes have been identified in the brains of vertebrates [30–36]. Although sex differences in the brain are often presumed to be a consequence of gonadal sex, rather than the cause [37], sex differentiation in the brain is a highly complicated process in lower vertebrates. Previous studies have suggested that gonadal fate may be affected by the brain. For example, forebrain transplants between male and female Japanese quail embryos, before sexual differentiation, disrupted testis function [38, 39]. Perceived social changes can also induce sexual transitions via intersection of the hypothalamic-pituitary-interrenal pathway and hypothalamic-pituitary-gonad pathway before sex differentiation in some sequentially hermaphroditic coral reef fish [40]. In zebrafish (*Danio rerio*), luteinizing hormone beta subunit (*lhβ*)/follicle stimulating hormone beta subunit (*fshβ*) double knock out resulted in all male fish, whilst gene disruption of *fshr*, but not *lhcgr*, resulted in masculinization into males and a complete failure of follicle activation [41, 42]. Until now, unlike gonadal sex differentiation, the mechanisms that underlie the sexual differentiation of the brain have not been completely defined. Identification of genes related to sex differentiation in the brain may facilitate studies of gene interaction between the gonads and brain, which control sex differentiation. However, few studies have focused on gene expression changes in the brain during the process of sex differentiation, particularly during the process of sex steroid hormone-induced feminization or masculinization in fish species such as the *T. rubripes* [29].

We previously reported the sexually dimorphic expression profile of genes in *T. rubripes* brain [43]. In this study, transcriptomic analysis of brains from the control and E_2_-treated groups was then performed. This study aimed to identify target genes and pathways that are involved in the development of *T. rubripes* brains and that responded to E_2_ administration. The data may provide new insights into the mechanism of sex differentiation in the brain and may indicate how estrogen affects gene expression in the brain.

## Results

### Histological evaluation of gonadal development

**Fig. 1:**
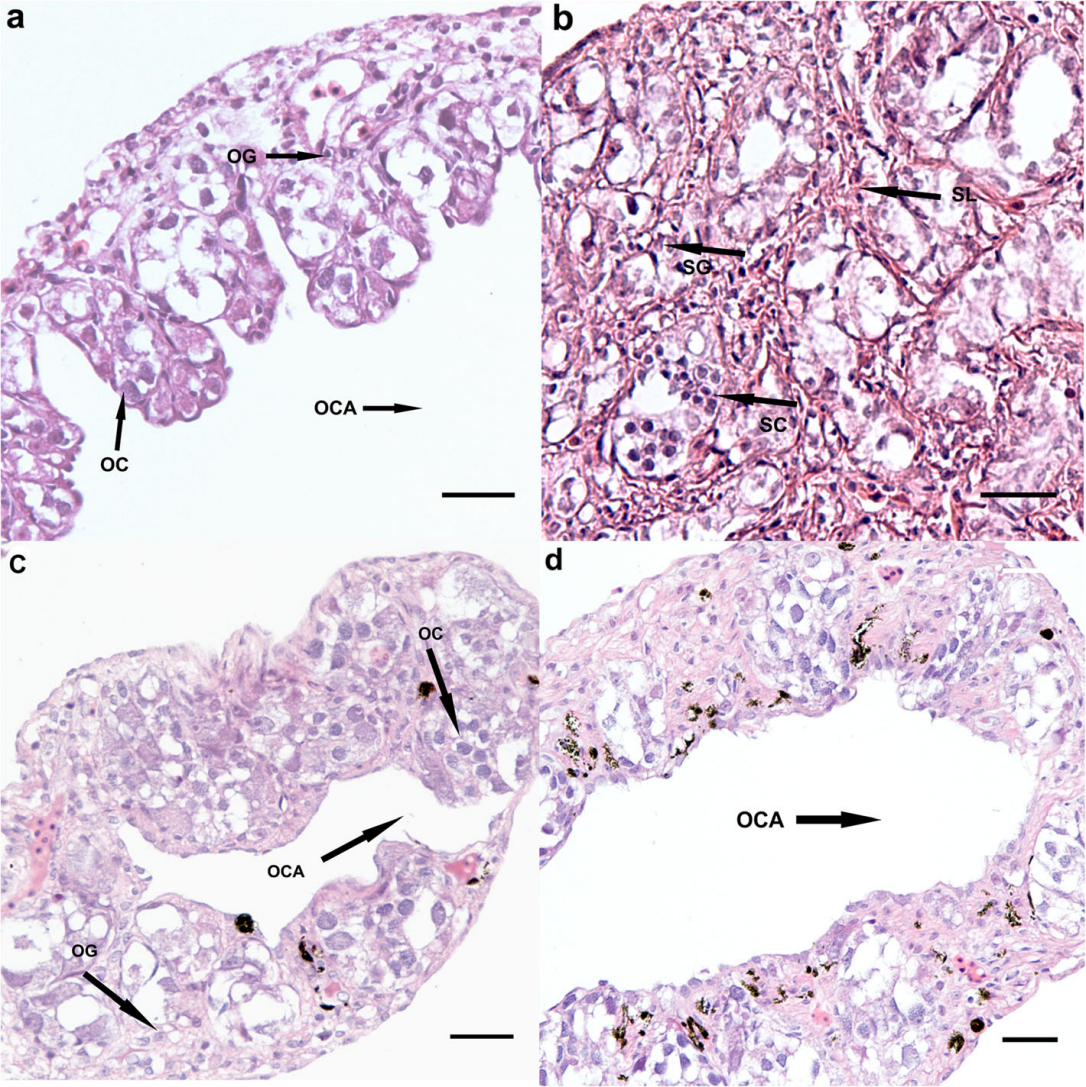
Hematoxylin-eosin stained gonad development sections from *Takifugu rubripes*. (**a**) C-XX, control group XX (**b**) C-XY, control group XY; (**c**) E_2_-XX, E_2_-treated XX (**d**) E_2_-XY, E_2_-treated XY. OG, oogonia; OC, oocyte; OCA, ovarian cavity; DOC, deformed ovarian cavity; SL, spermatogenic cysts; SG, spermatogonia; SC, spermatocyte. Scale bar, 30 μm

Figure 1 shows the results of the histological analysis of the gonads. As reported by Yan et al., sex reversed larvae were not observed in the control groups [22]. Gonads from the C-XX group occupied the ovarian cavities, which were filled with a small number of oocytes and a large number of oogonia, closely arranged on the oviposition plate. Gonads from the C-XY group were filled with spermatogenic cells at different developmental stages (Fig. 1a and b). However, gonads from the E_2_-immersed group were smaller than those from the control group. In addition, exposure to E_2_ obviously induced the feminization of testes, and a deformed ovarian cavity was observed in all E_2_-treated XY torafugu (Fig. 1c and d).

### Illumina sequencing and mapping, and identification of global DEGs, in response to E_2_ treatment

528,862,430 total reads were obtained from all library. After raw reads filtering, 43,622,952 (C-XX_1), 44,436,616 (C-XX_2), 42,850,608 (C-XX_3), 42,851,086 (C-XY_1), 45,072,134 (C-XY_2), 45,005,800 (C-XY_3), 43,442,122 (E-XX_1), 39,629,744 (E-XX_2), 48,253,210 (E-XX_3), 46,193,034 (E-XY_1), 42,700,218 (E-XY_2) and 44,804,906 (E-XY_3) clean reads were obtained from each library, respectively (Table 1).

**Table 1:** Summary statistics of the transcriptome sequencing and mapping in *Takifugu rubripes*

Sample	Raw data	Clean_reads	Total_map	Unique_map	Multi_map
C-XX_1	44,523,690	43,622,952	40,982,945(93.95%)	39,573,936(90.72%)	1,409,009(3.23%)
C-XX_2	44,523,690	44,436,616	41,718,759(93.88%)	40,266,124(90.61%)	1,452,635(3.27%)
C-XX_3	66,606,798	42,850,608	40,099,291(93.58%)	38,741,203(90.41%)	1,358,088(3.17%)
C-XY_1	43,978,518	42,851,086	39,724,413(92.7%)	38,336,011(89.46%)	1,388,402(3.24%)
C-XY_2	46,201,180	45,072,134	42,198,181(93.62%)	40,661,639(90.21%)	1,536,542(3.41%)
C-XY_3	46,409,586	45,005,800	42,144,064(93.64%)	40,678,707(90.39%)	1,465,357(3.26%)
E-XX_1	44,537,526	43,442,122	40,708,228(93.71%)	39,264,335(90.38%)	1,443,893(3.32%)
E-XX_2	40,345,586	39,629,744	37,253,090(94.0%)	35,927,359(90.66%)	1,325,731(3.35%)
E-XX_3	49,202,476	48,253,210	45,656,235(94.62%)	44,078,863(91.35%)	1,577,372(3.27%)
E-XY_1	47,529,109	46,193,034	43,403,167(93.96%)	41,703,371(90.28%)	1,699,796(3.68%)
E-XY_2	43,506,024	42,700,218	40,001,299(93.68%)	38,698,894(90.63%)	1,302,405(3.05%)
E-XY_3	45,728,118	44,804,906	42,008,134(93.76%)	40,598,629(90.61%)	1,409,505(3.15%)

**Fig. 2:**
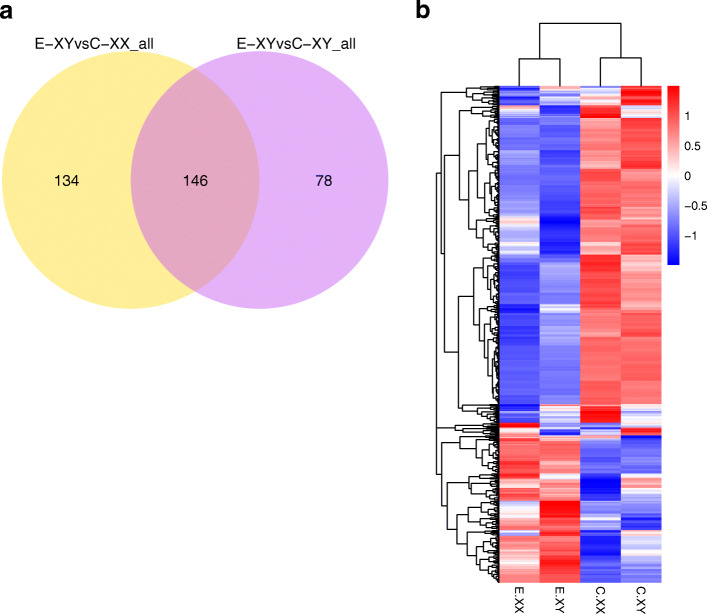
The DEGs observed between the control and E_2_-treated groups, based on FPKM units. Venn diagram (**a**) and Heat map (**b**). C-XX, control group XX; C-XY, control group XY; E_2_-XX, E_2_-treated XX; E_2_-XY, E_2_-treated XY

**Fig. 3:**
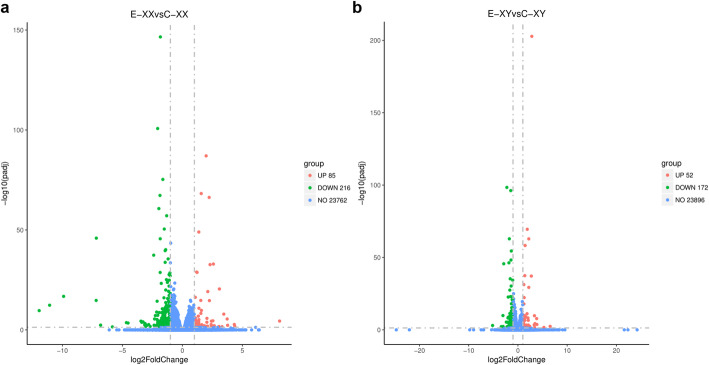
Volcano plot of differences in gene expression between control and E_2_-treated groups. (**a**) E-XX vs C-XX, (**b**) E-XY vs C-XY; Up-regulated genes (red), and down-regulated genes (green)

As shown in Figs. S1, only four DEGs were identified in the C-XY versus (vs) C-XX comparison, of which three DEG were up-regulated and one was down-regulated, such as aryl hydrocarbon receptor interacting protein-like 1 (*aipl1*), serine protease hepsin-like, and nucleoprotein TPR-like (Table 2). There were 411 DEGs between E_2_-treated (E-XX and E-XY) and Control (C-XX and C-XY) in total (Fig. 2). In the E-XX vs C-XX comparison, 301 DEGs were identified, of which 85 were up-regulated (Fig. 3 a). These included gonadotropin-releasing hormone 1 (*gnrh1*), cytochrome P450 aromatase (*cyp19a1b*), progesterone receptor (*pgr*), solute carrier family 6 (*slc6a20*) and cytochrome P450 1A1-like (*cyp1a1*). There were 216 down-regulated DEGs in this comparison, which included prolactin (*prl*), thyroid stimulating hormone (*tshb*), somatolactin-like (*sl*), glycoprotein hormones (*cga*) and pro-opiomelanocortin-like (*pomc*) (Table 2). Moreover, 224 DEGs were identified in the E-XY vs C-XY comparison, of which 52 were up-regulated (Fig. 3b), such as vitellogenin-2-like (*vtg2*), *pgr*, *gnrh1*, *cyp19a1b*, zona pellucida sperm-binding protein 4-like (*zp3*) and *cyp1a1*. 172 down-regulated DEGs were observed in this comparison. These included potassium channel (*kcnk18*), WD40 repeat-containing protein (*wd40*), basic helix-loop-helix family (*bhlhe41*) and forkhead box protein O1-a (*foxoa*) (Table 2). In the E-XX vs E-XY comparison, only 3 up-regulated DEGs were identified, including *hetc-domain*, *protein MAATS1-like* (*maats1*), and uncharacterized LOC105419364 (Table 2). In the E-XY vs C-XX comparison, 96 were up-regulated, such as *cyp19a1b*, *cyp1a1* and *pgr*. 184 down-regulated DEGs were observed, such as *bhlhe41*, *WD40* and *kcnk18* (Table S1).

**Table 2:** Selection of some of DEGs identified in C-XYvsC-XX, E-XXvsC-XX, E-XYvsC-XY, and E-XX vs E-XY

Gene name	log2 Fold change	Average FPKM		Description
**(C-XYvsC-XX)**		**C-XX**	**C-XY**	
*aipl1*	1.38	2.7	7.01	aryl hydrocarbon receptor interacting protein-like 1
LOC101065721	1.11	2.15	4.64	serine protease hepsin-like
LOC101063021	1.15	29.49	62.37	nucleoprotein TPR-like
**(E-XXvsC-XX)**		**E-XX**	**C-XX**	
*arntl*	1.37	18.73	7.26	aryl hydrocarbon receptor nuclear translocator-like
*bhlhe41*	− 2.06	11	45.96	basic helix-loop-helix family member e41
*cga*	−7.19	0.267	38.54	glycoprotein hormones alpha polypeptide
*cipc*	−1.96	15.72	44.78	CLOCK-interacting pacemaker-like
*ciart*	−1.33	6.45	16.25	circadian-associated transcriptional repressor-like
*cyp19b*	2.59	81.97	13.67	cytochrome P450 aromatase
*cyp1a1*	2.25	6.89	1.45	cytochrome P450 1A1-like
*gnrh1*	3.45	12.83	1.168	gonadotropin-releasing hormone 1
*per1*	−1.63	9.27	28.66	period circadian clock 1
*per3*	−1.31	10.26	25.51	period circadian clock 3
*pgr*	2.55	1.35	0.23	progesterone receptor
*prl*	−11.94	0.02	86.17	prolactin
*pomc*	−7.17	5.77	832.18	pro-opiomelanocortin-like
*pou1f1*	−4.66	0.053	1.37	POU class 1 homeobox 1
*tshb*	−11.07	0	52.91	thyroid stimulating hormone beta
*sl*	−9.9	0.04	38.65	somatolactin-like
*slc6a20*	2.12	8.82	2.03	solute carrier family 6 member 20
**(E-XYvsC-XY)**		**E-XY**	**C-XY**	
*bhlhe41*	−2.25	10.48	49.91	basic helix-loop-helix family member e41
*cyp19b*	2.82	90.7	12.84	cytochrome P450 aromatase
*cyp1a1*	1.84	5.66	1.58	cytochrome P450 1A1-like
*gnrh1*	3.35	17.5	1.72	gonadotropin-releasing hormone 1
*kcnk18*	−3.05	0.51	4.2	potassium channel two pore domain subfamily member 18
*per3*	−1.35	10.59	27.02	period circadian clock 3
*pgr*	3.81	1.51	0.11	progesterone receptor
*vtg2*	6.54	0.72	0.01	vitellogenin-2-like
*Wd40*	−2.89	3.19	23.56	WD40 repeat-containing protein SMU1-like
*zp4*	2.11	3.46	0.8	zona pellucida sperm-binding protein 4-like
**(E-XXvsE-XY)**		**E-XX**	**E-XY**	
*hetc*	8.77	2.78	0	HETC (ubiquitin-transferase)
*maats1*	1.18	5.6	2.41	protein MAATS1-like
*LOC105419364*	6.56	1.88	0	uncharacterized LOC105419364

### GO enrichment analysis of DEGs

**Fig. 4:**
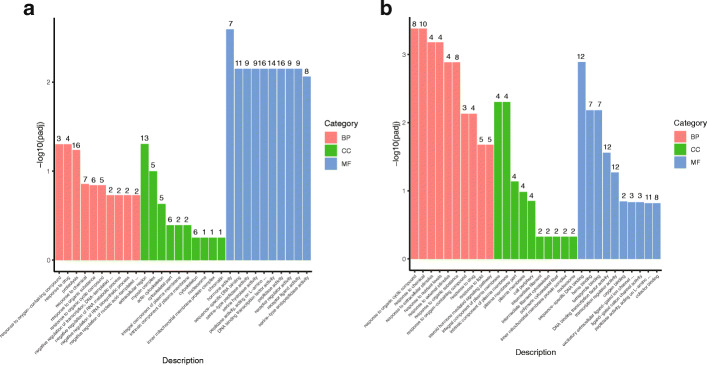
Gene ontology (GO) enrichment of DEGs for E-XX vs C-XX (**a**), and E-XY vs C-XY(**b**)

In the C-XY vs C-XX, E-XX vs C-XX and E-XY vs C-XY comparisons, genes were mainly enriched in biological processes, followed by molecular function and cellular component GO terms (Fig. S2, 4). In the C-XY vs C-XX comparison, the DEGs were mainly significantly enriched in microtubule-based movement and movement of cell or subcellular component in the biological process category. In the molecular function category, they were enriched in serine-type exopeptidase activity and exopeptidase activity (Fig. S2). In the E-XX vs C-XX comparison, DEGs were mainly enriched in response to oxygen-containing compound, response to drug and proteolysis, for biological process, in hormone activity, sequence-specific DNA binding and serine-type peptidase activity, for molecular function, and in calcium ion binding, myosin complex and actin cytoskeleton, for cellular component. The up-regulated genes were mainly clustered in proteolysis, for the biological process category, extracellular region, for the cellular component category, and hormone activity, for the molecular function category. The down-regulated genes were mainly clustered in cell cycle arrest, for the biological process category, myosin complex, for the cellular component category, and protein kinase regulator activity, for the molecular function category (Fig. 4a). In the E-XY vs C-XY comparison, response to oxygen-containing compound, response to chemical and response to extracellular stimulus were highly represented for the biological process category. Integral component of plasma membrane, intrinsic component of plasma membrane and plasma membrane part were highly represented for the cellular component category. Sequence-specific DNA binding, heme binding and tetrapyrrole binding were highly represented for the molecular function category. The up-regulated genes were mainly clustered in response to extracellular stimulus and response to nutrient levels, in the biological process category, integral component of plasma membrane, for the cellular component category, and sequence-specific DNA binding, for the molecular function category. The down-regulated genes were clustered in lipid transport and lipid localization in the biological process category (Fig. 4b).

### KEGG enrichment analysis of DEGs

**Fig. 5:**
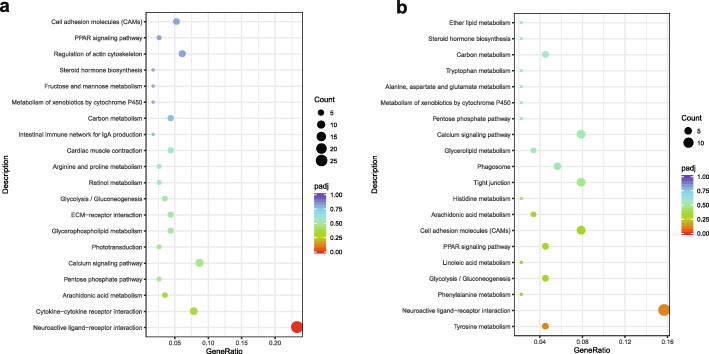
KEGG enrichment analyses of DEGs for E-XX vs C-XX (**a**), and E-XY vs C-XY (**b**)

The most enriched KEGG pathways in the E-XX vs C-XX comparison were neuroactive ligand-receptor interaction, arachidonic acid metabolism, cytokine-cytokine receptor interaction and the calcium signaling pathway. The KEGG pathways most enriched by down-regulated DEGs were neuroactive ligand-receptor interaction, steroid hormone biosynthesis, retinol metabolism, calcium signaling pathway and GnRH signaling pathway. Eight pathways, which included neuroactive ligand-receptor interaction, notch signaling pathway, cytokine-cytokine receptor interaction, PPAR signaling pathway, steroid biosynthesis, calcium signaling pathway, metabolism of xenobiotics by cytochrome P450 and GnRH signaling pathway, were the most enriched by up-regulated DEGs (Fig. 5a). In the E-XY vs C-XY comparison, the most enriched KEGG pathways were tyrosine metabolism, phenylalanine metabolism, arachidonic acid metabolism and linoleic acid metabolism. The pathways most enriched by down-regulated DEGs were steroid hormone biosynthesis, retinol metabolism, PPAR signaling pathway, carbon metabolism, metabolism of xenobiotics by cytochrome P450, calcium signaling pathway and neuroactive ligand-receptor interaction. The tyrosine metabolism, phenylalanine metabolism, arachidonic acid metabolism, linoleic acid metabolism, alpha-linolenic acid metabolism, steroid biosynthesis, histidine metabolism, metabolism of xenobiotics by cytochrome P450, tryptophan metabolism and calcium signaling pathways were those most enriched by up-regulated DEGs (Fig. 5b).

### qPCR

**Fig. 6:**
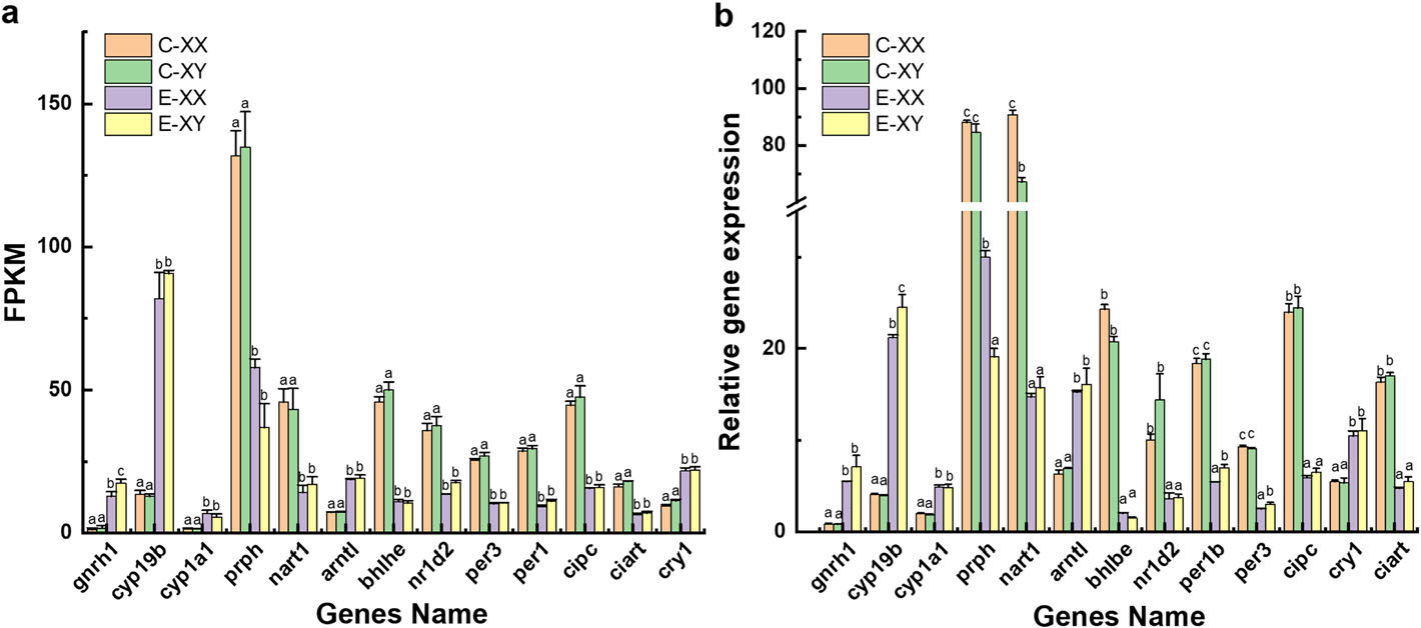
The expression level of *gnrh1*, *cyp19a1b*, *cyp1a1*, *prph*, *nart1*, *arntl1a*, *bhlbe*, *nr1d2*, *per1b*, *per3*, *cipc*, *cry1* and *ciart* in the *T. rubripes* brain after E_2_ treatment. FPKM (reads per kb per million reads) of obtained by RNA-seq (**a**). The relative expression levels of genes obtained by qPCR (**b**). C, Control group; E_2_, E_2_-treated group. Each value represents the mean ± SD of three measurements. One-way ANOVA (*p*-value < 0.05) was used for analysis

The qPCR analysis was used to verify RNA-Seq results (Fig. 6). In the control group, no significant difference in mRNA level of *gnrh1*, *cyp1a1* and *cyp19a1b* was found between the XY and XX groups (*p* > 0.05). In the E_2_ treatment group, the expression of *gnrh1*, *cyp1a1* and *cyp19a1a*, in XX and XY larvae, was significantly higher than in the control group (*p* < 0.05). The level of *cyp19a1b* in the E_2_-treated XY group was significantly higher than in the E_2_-treated XX group. In the control group, no significant difference was observed in the expression level of *prph*, *per1b, per3, cipc* and *ciart*, between XY and XX larvae, whilst the level of *nart1* was significantly lower in XY larvae than in XX larvae (*p* < 0.05). The expression levels of *bh1be, nr1d2, per1b, per3, cipc* and *ciart,* in E_2_-treated larvae brains, were significantly lower than in the control group (*p* < 0.05), whilst the expression levels of *arntl1a* and *cry1* were significantly higher than in the control group (*p* < 0.05).

## Discussion

To date, the use of E_2_ for gender control has been reported in at least 56 bony fish genera from 24 families. These fish include rainbow trout (*Oncorhynchus mykiss*), fighting fish (*Betta splendens*) and tilapia (*Oreochromis niloticus*) [44–49]. In *T. rubripes*, previous studies have also found that treatment with E_2_, during early sex differentiation, can induce feminization [8, 48, 49]. In our previous study, by E_2_-treatment (100 μg/L/time, 2 h/time, 1 time/day, from 25 dah to 55 dah), feminization of XY *T. rubripes* was induced, and the results of the transcriptomic analysis of gonads showed that a large number of DEGs and pathways involved in the process of feminization of XY *T. rubripes* [22]. For example, the expression of *cyp19a1a*, *foxl2*, *gsdf*, *dmrt1*, *cyp11a1*, *cyp17a1*, *hsd3b1*, *hsd17b1* and *cyp11c1* changed dramatically in the gonad when torafugu were treated with E_2_. In this study, we analyzed the brain transcriptome in order to better clarify the effects of estrogen on gonadal differentiation. The number of DEGs (eight DEGs at 80 dah) identified in the brain in the C-XX vs C-XY comparison was fewer than that identified at 30 dah (250 DEGs) and 40 dah (499 DEGs), in our previous study [43]. This could be attributed to the use of different differentiation stages of the torafugu that were employed in the two transcriptomic analyses. In addition, more DEGs were identified in the brain in both the E-XX vs C-XX comparison (514 DEGs) and E-XY vs C-XY comparison (362 DEGs). This suggests that the exogenous E_2_ triggered significant alteration of gene expression profiles in the brains of both XX and XY torafugu.

Various effects of exposure to E_2_ have been observed in teleosts. These effects include changes in brain development, endocrine regulation, gonadal development, growth, bone development, rhythm, feeding behavior and absorption, which are closely related to fish brain modulation [50, 51]. Aromatase (CYP19A1) catalyzes the biosynthesis of estrogens from androgens. In contrast to most mammalian species, which possess a single *Cyp19* gene, most teleosts possess duplicated copies of *cyp19a1*, namely *cyp19a1a* and *cyp19a1b* [52, 53]. The *cyp19a1a* gene is predominantly expressed in the ovary, while *cyp19a1b* is predominantly expressed in the brain and is critical for E_2_ biosynthesis [53, 54]. After E_2_ treatment, the expression level of *cyp19a1b* was up-regulated in the both XX and XY torafugu brain, which indicates that the E_2_ synthesized in the brain can be influenced by circulating E_2_ levels. The up-regulation of *cyp19a1b* gene expression by estrogenic compound administration also observed in zebrafish [55, 56]. In silico analyses of the torafugu *cyp19a1b* promoter have identified putative ERE binding sites (5′-TGACC-3′, 5′-GGTCAG-3′), similar to those identified in stickleback and medaka [57]. Thus, ERE may be necessary for E_2_-regulated *cyp19a1b* expression in torafugu.

The GnRH neurons are the principal output neurons from the hypothalamus and control reproduction [58]. Three paralogous forms of these neurons exist, termed GnRH1, GnRH2 and GnRH3 [59]. Pulsatile secretion of GnRH1 is essential for reproduction in all vertebrates. It induces follicle-stimulating hormone (FSH) and luteinizing hormone (LH) secretion in the pituitary, which triggers gonadal steroidogenesis [60]. In *T. rubripes*, the levels of *gnrh1* expression in the GnRH signaling pathway were up-regulated in the XX and XY brain. The use of E_2_ has also been shown to induce *gnrh1* up-regulation in other vertebrates [58–60]. In vivo experiments in mice showed that E_2_ rapidly acts as a hormone-activated transcription complex, to increase GnRH1 neuronal activity via ER [61]. However, ERs have not been identified as DEGs between our control and E_2_ treatment groups. This may be due to the limitation of the transcriptomic analysis being performed at a coarse anatomical scale (such as the whole brain). Thus, we cannot exclude the possibility that the up-regulation of *gnrh1*, by exogenous estrogen, occurs via ER in the torafugu brain.

In addition, the progesterone receptor (*pgr*) was up-regulated in the both XX and XY torafugu brain after E_2_ treatment. Like other members of the steroid receptor superfamily, progesterone receptors are vertebrate intracellular, ligand-inducible transcription factors [62] that are activated in the absence of their ligands by alterations in phosphorylation status [63]. Estradiol exerts positive or negative feedback on the hypothalamic-pituitary system [64–66]. Bashour et al., (2012) found that progesterone can act directly on GnRH neurons, through Pgr [66], and McCartney et al., (2009, 2010) found that the progesterone-sensitive mechanism is influenced by gonadal steroids [67, 68]. In rats, a previous study indicated that Pgr is a downstream mediator of the estradiol/ERα action in kisspeptin neurons [69, 70]. Therefore, *pgr* may be involved in the mediation of E_2_-induced *gnrh1* expression in the torafugu brain. In this study, we also found *vtg2* and *zp4* were expressed in brain of XY torafugu by transcriptome sequencing. Vitellogenin is synthesized in the liver of all oviparous taxa and transported in the blood to the ovary, which is the common yolk precursor protein [71]. The zona pellucida (ZP) is an extracellular glycoprotein matrix that surrounds all mammalian oocytes [72]. In teleost species, ZP genes are expressed either in liver under regulation of estrogen or in ovary [72]. However, why there is an extopic expression of those tow genes in torafugu brain and how they involved in the process of sex differentiation needs to study in the future.

Our previous study also found that the body lengths of larvae in E_2_-treated groups were less than those in the control group and the survival rate of larvae was only 17%. It proved that E_2_ significantly inhibits growth, survival and gonad development in torafugu larvae [22]. The results were similiar to data from both tilapia [73] and rainbow trout [74], in addition to results from another study on torafugu [48]. However, the mechanisms that underlie the effects are not clear. In mammals, it has been demonstrated that there is a close interdependence among the factors that regulate growth and reproduction, which involve the interactions between multiple growth peptides and estrogens, with their receptors [75, 76]. In our transcriptome data, somatotropin (*st*), thyroid stimulating hormone beta (*tshb*), somatolactin-like (*sl*), prolactin (*prl*) and pou1f1 (*pit-1*), which related development and growth of teletost [77–91] were down-regulated in the XX and XY *T. rubripes* brain after E_2_ treatment. Therefore, E_2_ may down-regulate those genes, by which inhibiting fugu growth and gonad development. Circadian rhythm is essential for living organisms to regulate a wide array of behavior and physiology, such as sleep, activity, reproduction, feeding and endocrine functions [92]. It exists in most life forms, from unicellular bacteria to higher organisms [93]. Although the basic regulatory mechanisms and functions follow the same general design, the conservation of expression of genes involved in the circadian rhythm, throughout the kingdom, is limited [94]. Here, transcriptomic and analysis showed that the core regulators of gene expression in the XX and XY brain, involved in the circadian rhythm, were altered by E_2_-treatment in both sexes in torafugu. For example, *arntl1a* and *cry1* were up-regulated and *bhlbe*, *nr1d2*, *per1*, *per3*, *cipc* and *ciart* were down-regulated after E_2_ treatment. Recently, there has been increasing evidence to suggest that estrogens can alter circadian clock gene expression in mammals [95–100]. Therefore, the significant alteration of the expression levels of circadian clock genes indicates that estrogen may also interfere with the biological clock in torafugu.

The DEGs observed between the E_2_-treated and control groups in both sexes were significantly enriched in KEGG pathways such as neuroactive ligand-receptor interaction, calcium signaling pathway and cell adhesion molecules (CAMs). The neuroactive ligand-receptor interaction pathway comprised all ligands and receptors in the cell membrane for signal transduction [100]. Cell adhesion molecules are proteins located on the cell surface and are required for the assembly and interconnection of various cellular functions, maintenance of tissue integration and wound healing [101, 102]. Our results suggest that exogenous stimulating hormones can interfere with signal transduction. In the E-XX group, the cytokine-cytokine receptor interaction pathway was the most significantly affected, when compared with E-XY. Cytokines can act in the CNS as immunoregulators and neuromodulators during health and disease [103, 104]. During cytokine-cytokine interactions, convergence of signaling pathways and divergence of the cytokine signal to activate other cytokine systems are involved in synergistic activities [104]. In the olive flounder, all 11 pathways were enriched in the brains of E_2_-treated phenotypic females, for example, circadian rhythm, circadian entrainment, dopaminergic synapse, calcium signaling, glutamatergic synapse, long-term depression, and taste transduction pathways, etc. [29]. The circadian rhythm, calcium signaling and glutamatergic synapse pathways were also enriched in our study. These results suggest that cell adhesion, transport, circadian rhythm and the calcium signaling pathway may be affected by exposure to E_2_ in teleosts.

In conclusion, by using transcriptomic sequencing of XX and XY brains of torafugu larvae to show that many genes and pathways were altered by E_2_ exposure. The genes and pathways identified here will help to elucidate the genetic basis behind the E_2_-induced feminization process. The data also open the possibility of investigating networks in the brain-pituitary-gonadal axis in torafugu.

## Materials and methods

### Animals

Twenty days after hatching (dah), torafugu larvae of 6.40 ± 0.1 mm body length were purchased from Dalian Fugu Aquatic Product Co., Ltd., Dalian, China.

### Treatment of *T. rubripes* larvae

Based on previous studies, treated fugu with 100 μg/L 17β-estradiol (E_2_) (2 h once a day) can induced feminization [22, 105], none of the treated XY fugu reverted to testes [105]. In the present study, after a short period of acclimatization (five days), 5700 larvae were randomly divided into two groups (control and E_2_-treated groups) and three replicates were created for each group (950 larvae/tank (approximately 100 L)). The oxygen level was maintained > 8 mg/L, the pH was maintained at 7.9–8.1, and salinity of 33 ppt and a temperature of approximately 20.5–21.5 °C. E_2_ powder of ≥99% purity (Sigma, St. Louis, MO, USA) was dissolved in 95% ethanol to form a solution for the experimental treatment. The E_2_ administration method was the same as described in our previous study [22]. The experiments were carried out at ~ 21.0 °C, under a natural photoperiod. Approximately 200 L water was changed after the two-hour E_2_ exposure**.**

### Tissue sampling

At the end of the experiment (55 days after treatment (dat)), larvae were anesthetized in ice water. Gonads to be used for histological analysis were dissected and fixed in 4% paraformaldehyde for 24 h. They were then stored in 70% ethanol. Sampling of brains was performed using 90 torafugu from each treatment (30 larvae per tank). Brains were stored individually in RNAlater reagent (Thermo Fisher Scientific, Baltics, USA), in a 1.5 mL plastic tube on ice. They were then snap-frozen in liquid nitrogen and stored at − 80 °C until RNA extraction and sequencing. For genetic sex verification, a piece of fin tissue sample was stored in a 1.5 ml tube containing 100% alcohol in a freezer at − 20 °C individually.

### Histological analysis, genetic gender verification and RNA preparation

**Fig. 7:**
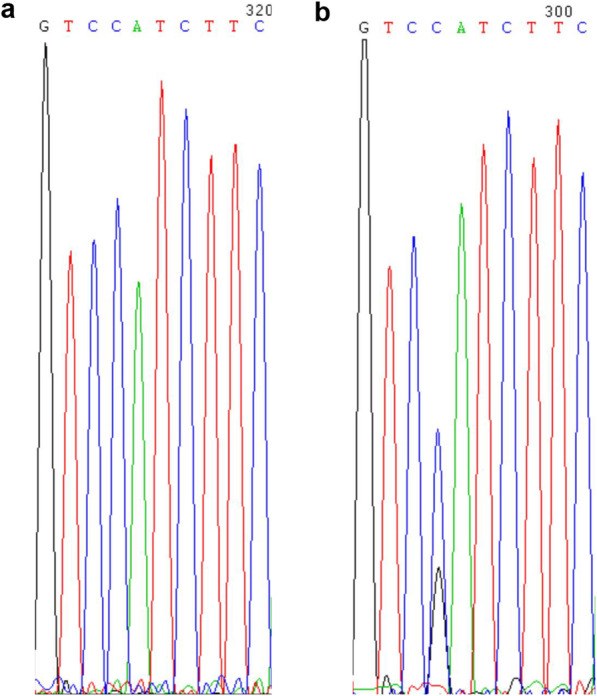
Sequence traces of *amhr2* from a female (left) and a male (right) fugu. The male is heterozygous at the non-synonymous SNP site that converts His384 codon into Asp384 codon

Histological analyses were conducted as described previously [19]. After histological observation, in order to identify E_2_-induced feminized torafugu, genomic DNA from the paraffin-block tissue was extracted in accordance with the manufacturer’s instructions (TIANamp FFPE DNA kit, Tiangen, China). For RNA-seq and quantitative (qPCR), genetic sex identification was also performed before RNA extraction. DNA was extracted from the fin tissues using the TIANamp Marine Animals DNA kit (Tiangen, Beijing, China). Genetic gender verification for each larva was performed before RNA extraction from brains. The gender was verified using an SNP on *amhr2* gene exon 9, through PCR amplified a region containing exon 9 and flanking introns using primers SD3exon8F (5′-CAGATGCACACAAACCACCT-3′) and SD3exon10R (5′-TCCCAGTGTTGCG GTATGTA-3′). Previous studies have demonstrated there is a perfect concordance between the SNP genotype and phenotypic sex [15, 18, 19]. As shown in (Fig. 7), the genotype of males was C/G (XY) and that of females was C/C (XX). After sex verification, total RNA from brain sample from each individual was prepared in accordance with a previously described protocol [19].

### RNA-Seq

Total RNA concentration was measured with a NanoDrop ND-1000 spectrophotometer (Thermo Scientific, Wilmington, DE, USA) and RNA integrity was assessed with an Agilent 2100 Bioanalyzer (Agilent Technologies, Santa Clara, CA, USA). Libraries were prepared as follows; for each replicate, a pool of 6 μg of RNA from genetic XX or XY torafugu (1 μg of purified brain RNA from each individual, six individuals were mixed together) was regarded as a single sample and 1 μg of RNA per sample was used as input material. Twelve sequencing libraries, which included the control XX (C-XX_1, C-XX_2, C-XX_3), control XY (C-XY_1, C-XY_2, C-XY_3), E_2_-treated XX (E-XX_1, E-XX_2, E-XX_3), and E_2_-treated XY (E-XY_1, E-XY_2, E-XY_3), were generated using a NEBNext Ultra RNA Library Prep kit for Illumina (NEB, Ipswich, MA, USA) [41]. The products were purified using an AMPure XP system (Beckman Coulter, Beverly, USA) to obtain a region of approximately 250 to 300 bp. The library preparations were conducted as previously described [19].

The reference genome and gene model annotation file were downloaded directly from NCBI (ftp://ftp.ncbi.nlm.nih.gov/genomes/all/GCF_000180615.1_FUGU5) and Hisat2 v2.0.5 was used for the alignment analysis of clean data from each library. The most common method, fragments of kilobase per exon model per million reads mapped (FPKM) was used to calculate gene expression levels. Differential expression analysis for the control or E_2_-treamented groups was conducted using the DESeq2 R package. The *p*-values were adjusted in accordance with methods that have been reported previously [42]. The threshold for significant differential expression was a *p*-value of 0.05 and log2 (fold-change) of 1 [106]. Subsequently, gene ontology (GO) and Kyoto Encyclopedia of Genes and Genomes (KEGG) enrichment analyses were performed to categorize differentially expressed genes (DEGs). The GO terms and pathways with a corrected *p-*value of less than 0.05 were considered significantly enriched.

### qPCR verification

The *gnrh1*, *cyp19a1b*, *cyp1a1*, *prph*, *nart1*, *arntl1a*, *bhlbe*, *nr1dd2*, *per1b*, *per3*, *cipc*, *cry1* and *ciart* genes were randomly selected for RNA-seq validation by qPCR, using an Applied Biosystems 7900 HT Real-Time PCR system, as described previously [18]. The reference gene used for the qPCR analysis was *β-actin*. Primers for the reference gene and other genes selected for validation were designed using the Primer Premier 5.0 program (Table 3). The relative expression of genes was calculated using the 2^−ΔΔCT^ method. Data are expressed as the mean ± SEM. Statistical significance analysis between the treatment and control groups was conducted using one-way ANOVA (*p*-value < 0.05) in the IBM SPSS software.

**Table 3:** Primers used for qPCR of *β-actin* and sex-biased genes

Name	Primer	Sequence (5′˗3′)	Length (bp)
*β-actin*	Forward	CAGATGTGGATCAGCAAGCA	245
	Reverse	GCTGAAGTTGTTGGGCATTT	
*gnrh1*	Forward	GCTGGTCGGGAGTCTGATGT	155
	Reverse	AACCCAGAAGAGCGGAGGA	
*cyp19b*	Forward	AACAAGTACGGCAGCCTGG	153
	Reverse	TCCCTCCATCCCGATACACT	
*cyp1a1*	Forward	ATGGCACCGAGGTCAACAA	119
	Reverse	CAGGATTGCCAGGAAGAGGTA	
*prph*	Forward	AAGCCATAGGAAAGGAGAGGG	137
	Reverse	GCGGAAGGCAATCAGGTTA	
*nart1*	Forward	TTCCCACAATAACCAGCATCA	147
	Reverse	CACGCTTACACTTTCAGCAACA	
*arntl1a*	Forward	TCCTGTTTGTGGTCGGTTGT	181
	Reverse	CTCTCTCGTGGGGCTGTATCT	
*bhlbe*	Forward	GCGACGGCAAAGATAAAGATAC	200
	Reverse	CTGTCCCACGCTGCTTATTC	
*nr1d2*	Forward	CGCCCACATCAACAAGGA	187
	Reverse	ATGTGCGTAGGTGGGAGTGT	
*per1b*	Forward	CACCCTCAACGCACTCAAA	175
	Reverse	GTCGGTGTTTTTCAGGGTGTA	
*per3*	Forward	ACAATGGTTCCAGCGGTTAT	109
	Reverse	TGCGAGTCCTCCCACAGA	
*cipc*	Forward	ACAGGGTCAAAGGAAGGGTG	105
	Reverse	GTTGGTGATGCTGATGCTTGT	
*cry1*	Forward	AGGCGGGTGTAGAGGTCATT	110
	Reverse	GGTCTGGAAACGCTTGTAGGT	
*ciart*	Forward	CGCTCCCTCCAAGATTCCT	145
	Reverse	TGAGACGAGGGCACTTGTAGAG	

## Supplementary Information

**Additional file 1.**

**Additional file 2.**

**Additional file 3.**

## Data Availability

The data sets supporting the results of this article are available at the SRA database of NCBI (https://www.ncbi.nlm.nih.gov/) under project accession number PRJNA760675.
