# Supplementary figures and images for: Profile of gene expression changes during estrodiol-17β-induced feminization in the Takifugu rubripes brain
Source: BMC Genomics. 2021 Nov 24;22:851. doi: 10.1186/s12864-021-08158-0 (PMC8614003; doi:10.1186/s12864-021-08158-0)

**FigS1. Volcano plot of differences in gene expression in the control groups.**

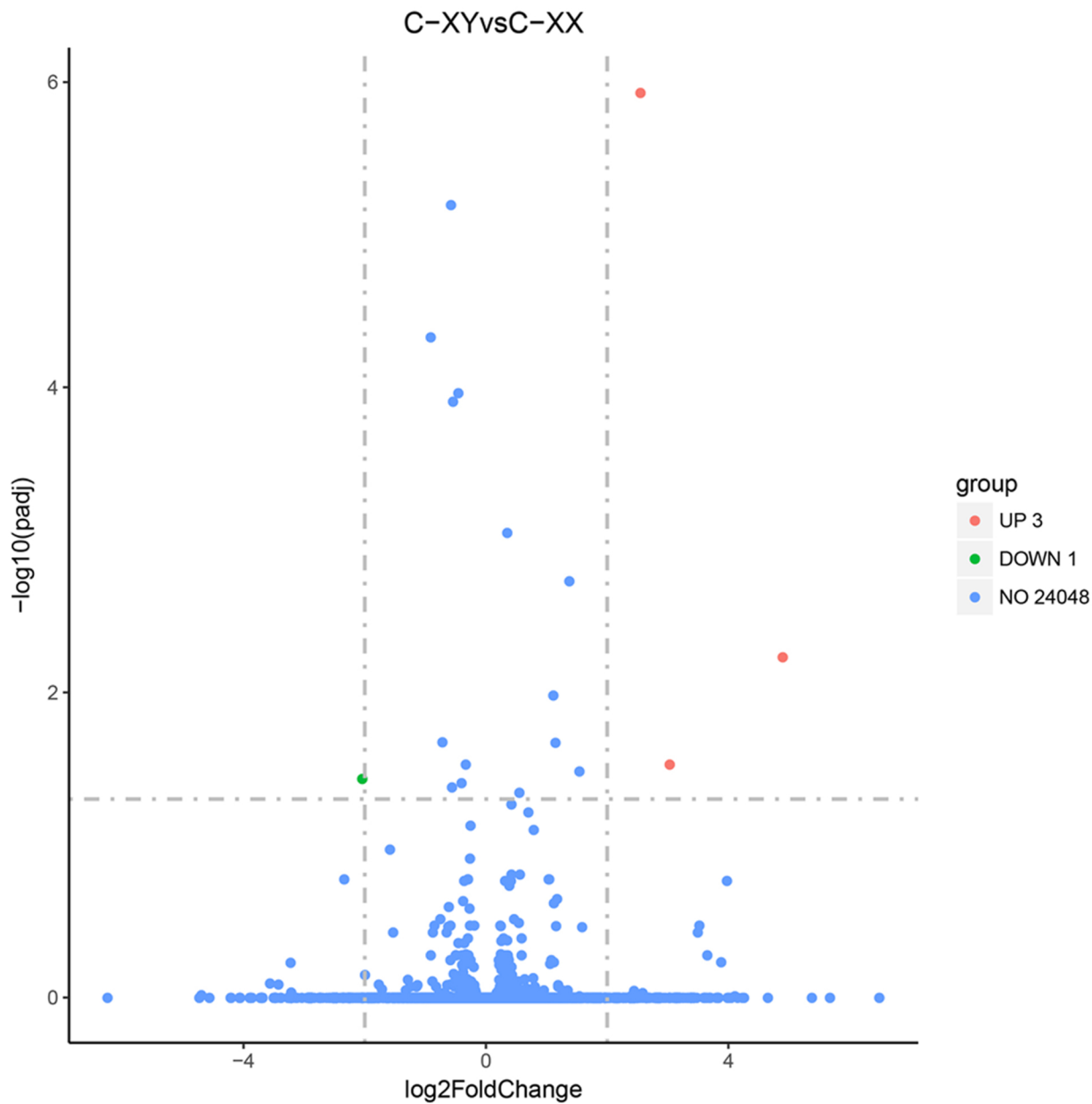

Supplement: Supplementary file 1 — Additional file 1. [file 12864_2021_8158_MOESM1_ESM.pdf]

**FigS2. Gene ontology (GO) enrichment of DEGs for C-XY vs C-XX.**

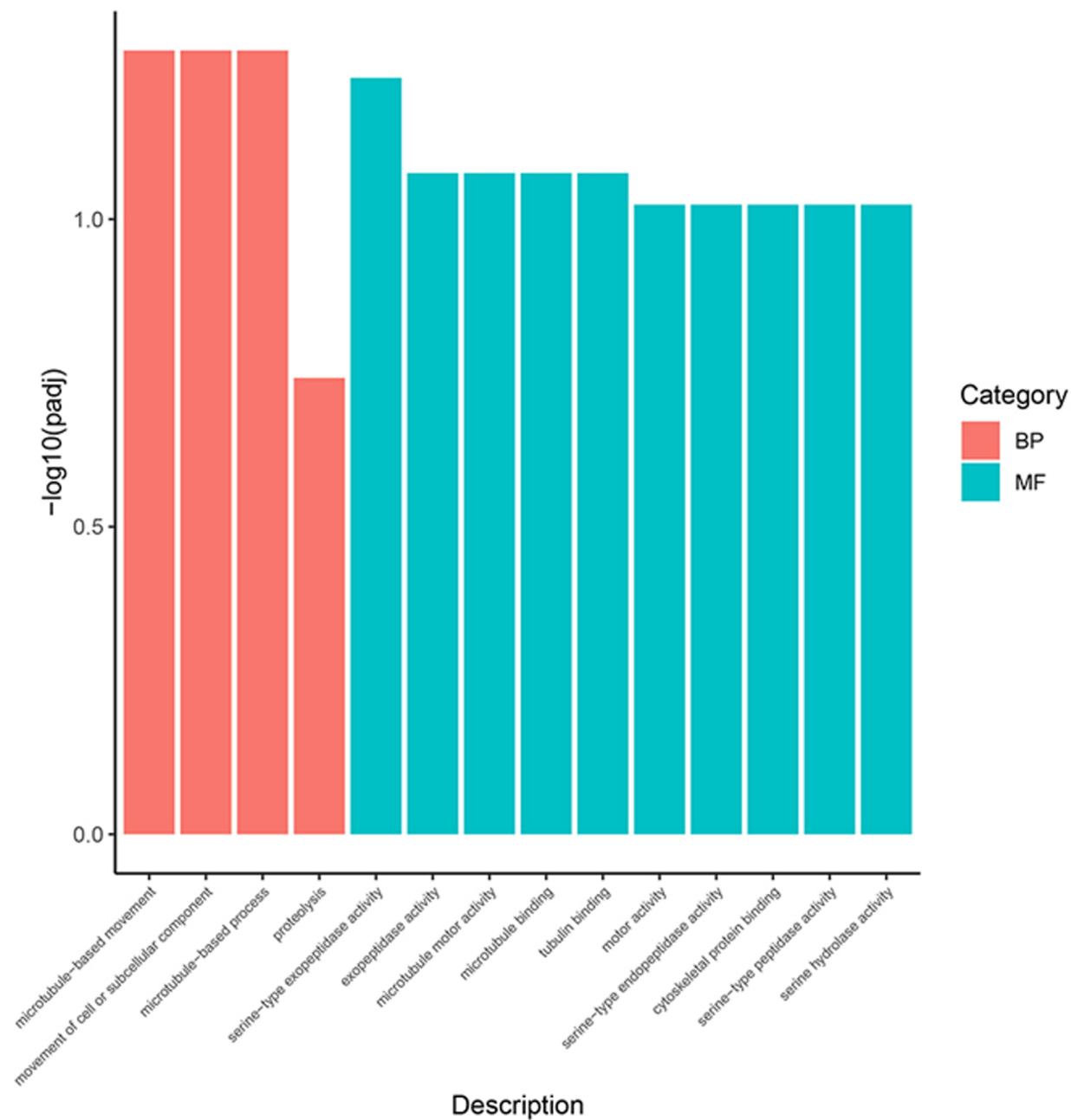

Supplement: Supplementary file 2 — Additional file 2. [file 12864_2021_8158_MOESM2_ESM.pdf]
