# Supplementary material for: Profile of gene expression changes during estrodiol-17β-induced feminization in the Takifugu rubripes brain
Source: BMC Genomics. 2021 Nov 24;22:851. doi: 10.1186/s12864-021-08158-0 (PMC8614003; doi:10.1186/s12864-021-08158-0)
Supplement: Supplementary file 3 — Additional file 3. [file 12864_2021_8158_MOESM3_ESM.docx]

| **Table S1. Selection of some of DEGs identified in E-XYvsC-XX** | | | | | |
| --- | --- | --- | --- | --- | --- |
| Gene name | log2 Fold change | Average FPKM | | | Description |
| **(E-XYvsC-XX)** | | **E-XY** | | **C-XX** |  |
| *cyp19a1b* | 2.73 | 90.7 | 13.67 | | cytochrome P450 aromatase |
| bhlhe41 | -2.13 | 10.48 | 45.96 | | basic helix-loop-helix family member e41 |
| per1 | -1.36 | 11.16 | 28.66 | | period circadian clock 1 |
| *cipc* | -1.49 | 16 | 44.78 | | CLOCK-interacting pacemaker-like |
| *WD40* | -2.76 | 3.19 | 21.52 | | WD40 repeat-containing protein SMU1-like |
| *arntl* | 1.41 | 19.24 | 7.26 | | aryl hydrocarbon receptor nuclear translocator |
| *cyp1a1* | 1.96 | 5.66 | 1.45 | | cytochrome P450 1A1-like |
| *pgr* | 2.71 | 1.51 | 0.23 | | progesterone receptor |
| *kcnk18* | -2.84 | 0.51 | 3.62 | | potassium channel two pore domain subfamily K member 18 |
| *gnrh1* | 3.9 | 17.5 | 1.17 | | gonadotropin-releasing hormone 1 |
| *zp4* | 3.4 | 3.46 | 0.33 | | zona pellucida sperm-binding protein 4-like |
